# Population-based hospitalization incidence of respiratory viruses in community-acquired pneumonia in children younger than 5 years of age

**DOI:** 10.1111/irv.12277

**Published:** 2014-09-03

**Authors:** Susan S Chiu, Pak-Leung Ho, Malik J S Peiris, Kwok Hung Chan, Eunice L Y Chan

**Affiliations:** a Department of Pediatrics and Adolescent Medicine, The University of Hong KongHong Kong SAR, China; b Department of Microbiology, The University of Hong KongHong Kong SAR, China; c Centre of Influenza Research, School of Public Health, The University of Hong KongHong Kong SAR, China

**Keywords:** Bacterial pneumonia, children <5 years, hospitalization, virus

*Please cite this paper as:* Chiu *et al*. (2014) Population-based hospitalization incidence of respiratory viruses in community-acquired pneumonia in children younger than 5 years of age. Influenza and Other Respiratory Viruses 8(6), 626–627.

To the editor:

Respiratory viral infections are common in young children but the extent of their association with community-acquired pneumonia remains unclear. Previous work on this issue was limited in testing only a limited number of viruses or that the findings not expressed as population-based incidence. This study addressed this by a retrospective analysis of the respiratory specimens collected from children aged <5 years with acute febrile respiratory symptoms who were admitted to two hospitals that together catered for 72·5% of all pediatric admissions on Hong Kong Island. Respiratory viruses were tested for by immunofluorescence (RSV, influenza A/B, parainfluenza types I–III, adenovirus) with turnaround time of several hours and results known to attending pediatricians at the time, and culture and PCR (results not known to pediatricians) in nasal secretions. The study was approved by the Institution Review Board of Queen Mary Hospital/Hong Kong West Cluster.

A total of 441 children <5 years of age were admitted for an acute respiratory infection between 2002 and 2004 with 1-in-7-day systematic sampling. Sixty children, of whom 53 had a respiratory sample sent for testing, were given the discharge diagnosis of pneumonia by the attending pediatricians based on physical examination, CXR and laboratory investigation, and treated as bacterial pneumonia with a full course of beta-lactam antibiotic. The seven children who did not have an NPA were admitted around the time of the SARS outbreak. One child tested positive for SARS-CoV. Thirty-two (60·4%) children who were diagnosed of bacterial pneumonia by pediatricians and tested for respiratory virus had at least one virus detected. Seven children with pediatrician diagnosed bacterial pneumonia had two viruses detected: adenovirus and rhinovirus (RV) in 2, bocavirus and RV in 2, respiratory syncytial virus (RSV) and adenovirus in 1, RSV and bocavirus in 1, and RSV and RV in 1. There was no statistically significant difference in detection frequency in those <2 years and 2 to <5 years of age for any virus. (data not shown). Nineteen (31·6%) children were diagnosed and treated for superimposed bacterial pneumonia after the availability of rapid virology test results, while a viral association was not suspected in another 22% of children treated for bacterial pneumonia. There was no statistically significant difference in clinical severity between children with bacterial pneumonia with or without associated viral detected although there was a trend that those who had a virus detected were more likely to require supplementary oxygen (data not shown).

We delineate that the incidence of hospitalization for bacterial pneumonia with at least one virus identified to be 232·7 (95% CI: 94·1, 479·6) and 528·9 (95% CI: 342·5, 780·5) per 100 000 children <2 years and 2–<5 years, respectively. (Table 1) The rates were highest in association with RV and RSV with the population-based hospitalization incidence higher in the older children when compared to children <2 years of age.

**Table 1:** Mean annual age-specific rate of virus associated bacterial pneumonia per 100 000 children (95% CI) in Hong Kong Island (2002–04)

	<2 years	2–<5 years	Overall <5 years
Population	14 525	22 818	37 343
Bacterial pneumonia	365·6 (182·5, 653·8)	1036·7 (766·9, 1370·3)	775·7 (591·8, 998·3)
At least one virus detected	232·7 (94·1, 479·6)	528·9 (342·5, 780·5)	413·7 (283·1, 584·2)
RV	99·7 (20·3, 291·5)	190·4 (87·2, 361·6)	155·1 (80·0, 271·1)
RSV	99·7 (20·3, 291·5)	148·1 (59·9, 305·3)	129·3 (61·9, 237·6)
Influenza A or B	0 (0, 122·6)	84·6 (22·8, 216·9)	51·7 (14·0, 132·5)
Adenovirus	33·2 (0·7, 185·1)	63·5 (12·9, 185·5)	51·7 (14·0, 132·5)
Parainfluenza types I-III	0 (0, 122·6)	21·2 (0·4, 117·8)	12·9 (0·3, 72·0)
HMPV	0 (0, 122·6)	21·2 (0·4, 117·8)	12·9 (0·3, 72·0)
HBoV	33·2 (0·7, 185·1)	84·6 (22·8, 216·9)	64·6 (20·8, 150·7)
HCoV	0 (0, 122·6)	21·2 (0·4, 117·8)	12·9 (0·3, 72·0)

RV, rhinovirus; RSV, respiratory syncytial virus; HMPV, human metapneumovirus; HBoV, human bocavirus; HCoV, human coronavirus (229E, OC43, NL63, and HKU1).

There was a high incidence of viral detection in children diagnosed and treated for bacterial pneumonia in Hong Kong. Studies from different countries in different host populations also documented rates of viral-bacterial coinfection in childhood with community-acquired pneumonia ranging from 16% to 66%.^1^ Although influenza infection is associated with very high hospitalization rates in children in Hong Kong,^2^ it accounted for a small proportion in this study of children with bacterial pneumonia. In summary, this study showed that respiratory viruses play a significant role in young children hospitalized and treated for presumed bacterial pneumonia, whether as co-infection or misdiagnosed as bacterial pneumonia, and documented the age-specific hospitalization incidences in children <5 years.

## Supporting Information

Appendix S1 Seasonal Distribution of Viral Detection.
