# Supplementary material for: Population-based hospitalization incidence of respiratory viruses in community-acquired pneumonia in children younger than 5 years of age
Source: Influenza Other Respir Viruses. 2014 Sep 3;8(6):626–7. doi: 10.1111/irv.12277 (PMC4262277; doi:10.1111/irv.12277)
Supplement: Appendix S1 — Seasonal Distribution of Viral Detection. [file irv0008-0626-sd1.docx]

Appendix S1.

Seasonal distribution of viral detection in children < 5 years hospitalized for bacterial pneumonia (2002-2004).

Figure.
